# Effectiveness of Robot Interventions for Cognitive and Psychological Outcomes among Older Adults with Cognitive Impairment: A Meta-Analysis

**DOI:** 10.3390/healthcare11162341

**Published:** 2023-08-19

**Authors:** Dabok Noh, Mi-So Shim

**Affiliations:** 1 College of Nursing, Eulji University, Seongnam 13135, Republic of Korea; daboknoh@gmail.com; 2 College of Nursing, Keimyung University, Daegu 42601, Republic of Korea

**Keywords:** dementia, meta-analysis, mild cognitive impairment, older adults, robotics, systematic review

## Abstract

This review was performed to evaluate the effects of robot interventions on cognitive and psychological outcomes among older adults with cognitive impairment. Three databases (PubMed, Embase, and Cochrane Central Register of Controlled Trials) were searched for studies published in English between January 2015 and August 2021. We included studies that involved older adults with cognitive impairment, interventions using robots, outcome measures related to cognitive and psychological status, and randomized controlled trials. Ten studies included in the systematic review, and nine studies derived from these ten articles were included in the meta-analyses. The meta-analyses revealed that robot interventions significantly decreased anxiety and agitation but exerted no significant effects on cognitive function, neuropsychiatric symptoms, and quality of life. The subgroup analyses according to robot types revealed that pet-type robot interventions reduced anxiety and agitation. In addition, subgroup analysis according to the intervention format of robot interventions found that individual intervention was effective for improving agitation, but a group-based intervention was effective for improving depression. We suggest using robot interventions to improve psychological outcomes such as anxiety and agitation; however, further research is needed to determine whether robot interventions affect symptoms such as cognitive function, neuropsychiatric symptoms, and quality of life.

## 1. Introduction

Owing to the increasing aging population, dementia has been recognized as a public health priority worldwide. Currently, the global prevalence of dementia has reached approximately 55 million people and is expected to nearly triple to more than 152 million people by 2050 [1]. Along with this rapid increase in prevalence, the health-economic cost of dementia is also expected to increase considerably [2].

Dementia is a degenerative neurocognitive disorder characterized by a decline in cognitive function, and its clinical features include cognitive impairment and clinically significant behavioral and psychological disturbances [3]. The behavioral and psychological symptoms of dementia, including aberrant motor behavior, depression, appetite and eating changes, agitation, anxiety, sleep disturbances, night-time behavior, delusion, irritability, and hallucination, affect patients’ quality of life and increase caregivers’ burdens [4]. Previous systematic reviews suggested that agitation and anxiety increased as the severity of dementia increased, and the prevalence of symptoms such as depression, agitation, and anxiety was as high as 20% or more for community-dwelling older adults with dementia [5]. Meanwhile, various symptoms such as depression, delusion, hallucinations, apathy, and sleep disorders appearing in patients with dementia are grouped into a cluster called neuropsychiatric symptoms, and these symptoms negatively affect the quality of life of patients with dementia [6].

Dementia can be preceded by mild cognitive impairment (MCI). MCI is a transition stage between the cognitive decline expected in usual aging and the cognitive decline in dementia [7]. According to a study by Mitchell and Shiri-Feshki [8], the rate of annual transition from MCI to dementia was 9.6% and 4.9% in specialist settings and general population settings, respectively. According to previous studies, it is estimated that 35–85% of older adults with MCI experience neuropsychiatric symptoms [9]. Depression, anxiety, and agitation were reported as the most common symptoms [9]. Although the effectiveness of non-pharmacological interventions for older adults with MCI on dementia prevention has not been fully proven, the rationale has been published that they reduce the risk of progression to cognitive decline or dementia [10]. Therefore, recent studies are attempting to identify the effects of interventions during the MCI period on the prevention of dementia and the maintenance of cognitive function.

The increased care burden for older adults with dementia has led to the development of robotic assistive technology. As a result of prior review of older adults’ experiences and perceptions of socially assistive robots, some older adults express negative attitudes or fears towards robots, but the older adults have relatively positive experiences about the function or usefulness of socially assistive robots [11]. Socially assistive robot technology was designed to meet the social and psychological needs of older adults through human–robot interactions [12]. Socially assistive pet-type robots can act as companions for older adults by imitating the appearances and behaviors of animals, whereas socially assistive humanoid robots have human-like shapes and features; both types of robots can interact with older adults [13]. Tanaka et al. [14] presented the effects of an intervention using a communication robot for older women and found that the intervention was effective in improving cognitive function, fatigue, and motivation. Additionally, Lee et al. reported that robot intervention for older adults with MCI effectively improved cognitive function and anxiety [15]. Thus, these robots have been used to deliver cognitive or psychosocial support for older adults.

Considering that research on socially assistive robots has increased, evaluating their effectiveness is of great importance. A recent literature review focused on humanoid robots and comprehensively described their effects on patients with dementia; however, a meta-analysis for quantitative synthesis was not conducted [16]. Other systematic reviews evaluated the effects of robot interventions through meta-analysis; however, the reviewed participants were restricted to patients with dementia [17,18]. Furthermore, a systematic review evaluated the effects of socially assistive robots on older adults with dementia or cognitive impairment through meta-analysis; however, the reviewed outcomes were restricted to three psychological outcomes: agitation, quality of life, and depression [19].

We aimed to evaluate the effects of interventions that use robots on cognitive and psychological outcomes among older adults with cognitive impairment. We sought to resolve the unreviewed issues in previous literature reviews. Given that robot interventions may be more beneficial for older adults with MCI because they can be more responsive to robots than those with severe cognitive impairment [20], we reviewed studies involving older adults with cognitive impairment, including dementia and MCI, regardless of the severity of cognitive impairment. Moreover, we also reviewed interventions that used robots without restrictions on the type of robot. Additionally, we evaluated the outcomes related to cognitive and psychological status without restrictions to identify which outcomes were examined in previous studies and to examine the effects of robot interventions through a meta-analysis. We then conducted subgroup analyses to evaluate effects according to robot types and intervention format because previous studies reported variations in user experiences linked to factors such as the robot’s appearance, design, and the specific tasks and methods employed when implementing robot interventions for older adults [11].

## 2. Material and Methods

### 2.1. Design

This systematic review and meta-analyses were performed according to the Cochrane Handbook for Systematic Reviews of Interventions version 6.2 [21] and reported according to the standards of the Preferred Reporting Items for Systematic Reviews and Meta-Analyses 2020 Statement [22].

### 2.2. Eligibility Criteria

The inclusion criteria were as follows: (a) studies that involved older adults with cognitive impairment, including dementia and MCI, regardless of severity; (b) studies that provided an intervention using robots; (c) studies that examined outcome measures related to cognitive and psychological status; and (d) randomized controlled trials (RCTs) or cluster randomized trials (CRTs) with a comparison group.

The exclusion criteria were as follows: (a) studies with samples including people without cognitive impairment; (b) studies that did not include outcome variables related to cognitive and psychological status; and (c) reviews, study protocols, conference abstracts, editorials, and observational studies.

### 2.3. Information Sources and Search Strategy

We searched three core databases (PubMed, Embase, and Cochrane Central Register of Controlled Trials) according to the Cochrane Handbook for Systematic Reviews of Interventions on 31 August 2021 [23]. We developed a search strategy by combining controlled vocabulary search terms [e.g., Medical Subject Headings (MeSH) and Emtree] and text words [e.g., cognitive impairment, mild cognitive, MCI, cognitive decline, memory impairment, memory decline, dementia, Alzheimer, dementia (MeSH), robot*, robotics (MeSH), randomized, controlled clinical trial, randomized controlled trial, placebo, clinical trials as a topic (MeSH), randomly, and trial]. Since 2015, experimental studies using robots to care for older people have increased [17,18]. Therefore, the search was limited to articles published in English from January 2015 to August 2021. Supplementary Table S1 presents search strategies for databases.

### 2.4. Selection Process

The search results across databases were merged using EndNote^®^ (EndNote X9.3.3, Thomson Reuters, NY, USA). The duplicates were electronically and manually removed. Subsequently, the eligibility of the remaining articles was reviewed based on titles and abstracts. After removing irrelevant articles, the full texts of the remaining articles were assessed to determine study eligibility. Two independent researchers screened the articles and resolved any discrepancies through discussions.

### 2.5. Data Extraction

Data extraction was performed using a standardized data extraction sheet. Two independent researchers performed data extraction, and discrepancies were resolved through discussions. The extracted data included authors; year of publication; country; characteristics of participants, such as age, sex, sample size, recruitment settings, and cognitive status; intervention characteristics, such as intervention method, intervention duration, intervention frequency, and time per each session; comparison condition; outcomes related to cognitive and psychological status; measurement tools; measurement assessment time points; results; and risk-of-bias data.

### 2.6. Risk-of-Bias Assessment

The risk of bias was assessed using version 2 of the Cochrane risk-of-bias tool for RCTs and CRTs [24,25]. Two independent researchers performed the risk-of-bias assessment. The tool for RCTs includes the following five domains: (a) randomization process, (b) deviations from the intended interventions, (c) missing outcome data, (d) measurement of the outcome, and (e) selection of the reported result. The tool for CRTs includes “timing of identification or recruitment of participants” as the sixth domain, along with the five aforementioned domains. Within each domain, there were signaling questions and response options. By using the answers to the signaling questions, judgments for each domain (i.e., “low risk of bias,” “having some concerns,” or “high risk of bias”) led to an overall risk of bias for each study. Based on discussions and consensus, disagreements between two independent researchers were resolved.

### 2.7. Data Synthesis

To assess the effectiveness of robot interventions in improving cognitive and psychological outcomes among older adults with cognitive impairment, we conducted separate meta-analyses according to the type of outcomes. For meta-analyses of continuous data, we extracted the means, standard deviations (SDs), and sample sizes at the post-test in the experimental and control groups or the mean changes, SD differences, and sample sizes in each group. These numerical data derived from the included studies were entered into Comprehensive Meta-Analysis (version 3; Biostat, Englewood, NJ, USA). We calculated Hedges’ *g* effect sizes and 95% confidence intervals (CIs). Given the existence of heterogeneity in the characteristics of the populations and interventions across the included studies, we applied DerSimonian and Laird’s random-effects approach. If the lower and upper limits of the 95% CI included zero, the effect size of the meta-analysis result is not statistically significant. We provided forest plots displaying effect estimates and 95% CIs for both individual studies and the meta-analyses. Heterogeneity was assessed using Q, I^2^, and Tau^2^ statistics. According to the Cochrane Handbook for Systematic Reviews of Interventions, a low *p*-value provides evidence of heterogeneity in the Q statistic with a *p*-value [26]. The I^2^ statistic result was interpreted as follows: 0–40% for possible unimportance, 30–60% for moderate heterogeneity, 50–90% for substantial heterogeneity, and 75–100% for considerable heterogeneity [26]. Additionally, subgroup meta-analyses were performed based on the type of robot and intervention format.

## 3. Results

### 3.1. Study Selection

**Figure 1:**
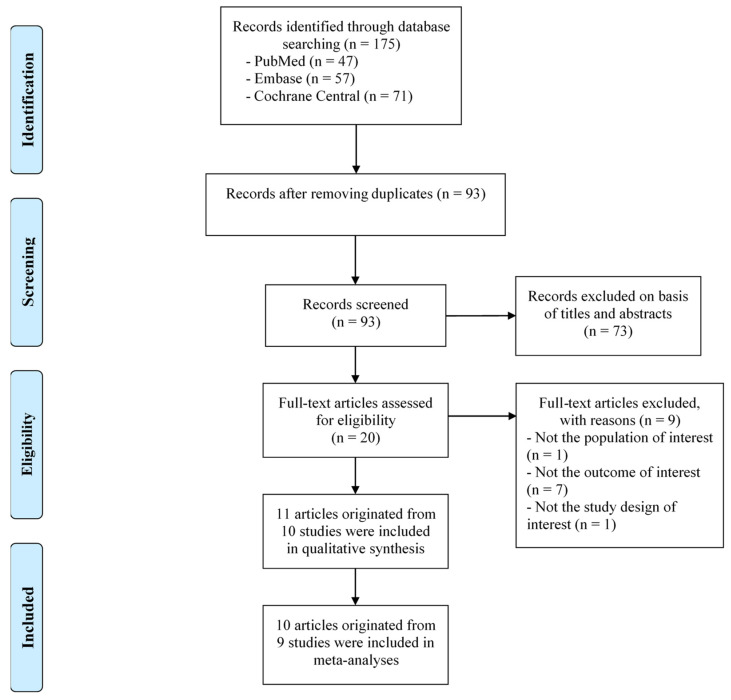
Flowchart presenting the study selection process.

Figure 1 shows a flow diagram of the study selection process. The initial search of the three databases identified 175 articles (i.e., PubMed = 47, Embase = 57, and Cochrane Central Register of Controlled Trials = 71), and 93 articles remained after 82 duplicates were removed. In the process of screening the articles’ titles and abstracts, 73 articles were excluded, and 20 articles were retained. Subsequently, during the assessment of the articles’ full texts, nine articles were excluded. The reasons for exclusion were as follows: not the population of interest [27], not the outcome of interest [28,29,30,31,32,33,34], and not the study design of interest [35]. Of the remaining 11 articles, 2 [36,37] originated from one study. Conclusively, 10 studies reported in 11 articles were included in this review, and 9 studies derived from the 10 articles reporting sufficient numerical data to calculate effect sizes were included in the meta-analyses.

### 3.2. Study Characteristics

Table 1 shows the detailed descriptions of the included studies. Most studies (n = 8) adopted an RCT design, whereas two adopted a CRT design. Most studies (n = 6) compared one experimental group with one control group, such as usual care, standard care, and no intervention. By contrast, four studies used three groups for comparison. Specifically, a study compared a robotic seal with a nonrobotic plush toy and usual care [38]; one study compared robot-assisted cognitive training with traditional cognitive training and no intervention [39]; and another study compared a robot seal with a real dog and a soft toy cat [40]. Furthermore, a study compared a humanoid robot, a pet robot, and standard care in the first phase and compared a pet robot, a real dog, and standard care in the second phase [41].

Ten studies, with a total of 1191 participants, were analyzed. Each study’s sample size ranged from 24 to 415 [15,20,21,22,24,25,26,27,28,29,30,31,32,33,34,35,36,37,38]. Among the 10 studies, 7 recruited participants from long-term care facilities [38,42,43,44] or nursing homes [36,37,40,41]. The remaining three studies recruited participants from a hospital [15], daycare centers [20], and a dementia center [39]. Regarding the cognitive status of the participants, most of the studies (n = 8) targeted individuals with dementia, one focused on those with MCI [15], and one study targeted those with MCI or subjective memory complaints [39]. The mean age of the participants ranged from 74.0 [15] to 87.2 years [42].

Based on their appearance, the robots were classified into pet-type, humanoid, and tabletop-type robots. Seven studies used a pet-type robot named PARO, which is a robotic pet resembling a baby harp seal [20,36,37,38,40,41,43,44]. Three studies used three humanoid robots called Kabochan [42], Sil-bot [39], and NAO [41]. Specifically, Valentí Soler et al. [41] compared a humanoid robot (NAO) and a pet-type robot (PARO) with a control group in the first phase. Only one study used a tabletop-type robot named Bomy [15].

Regarding intervention formats, five studies provided interventions using robots on an individual basis [15,38,40,42,44], and four studies conducted group programs [20,36,37,39,43]. In one study, group sessions were provided, and PARO was used in a personalized manner at home; therefore, the study was classified as using a group program [20]. One study employed group or individual sessions according to the cognitive function of the participants’ dementia: group sessions for participants with mild to moderate dementia and individual sessions for those with moderate to severe dementia [41].

### 3.3. Risk of Bias in Included Studies

Supplementary Figure S1 presents the risk-of-bias results. Among the ten included studies, three RCTs and one CRT had an overall risk of bias of “some concerns”, and five RCTs and one CRT had a high risk of bias. Four RCTs and one CRT had a high risk of bias because of blinding issues, and the deviations from the intended intervention were not balanced between groups. Three RCTs had a high risk of bias because of missing outcome variables and differences in the rates of attrition and dropout reasons between the groups. Additionally, one CRT had a high risk of bias owing to the recruitment of participants or the timing of identification because there was no information on whether all individual participants were identified and recruited prior to cluster randomization.

### 3.4. Postintervention Effects

**Figure 2:**
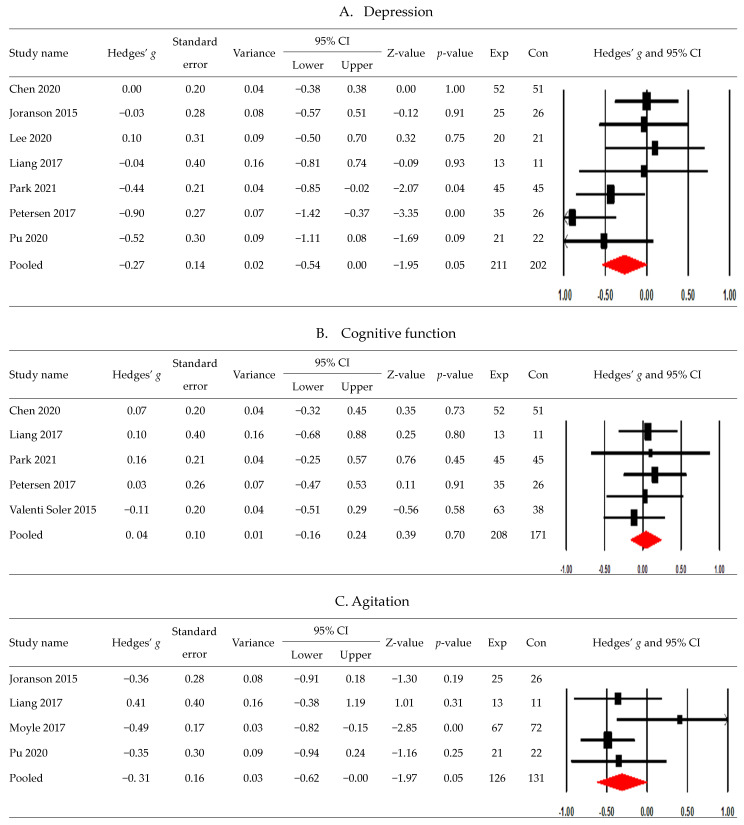

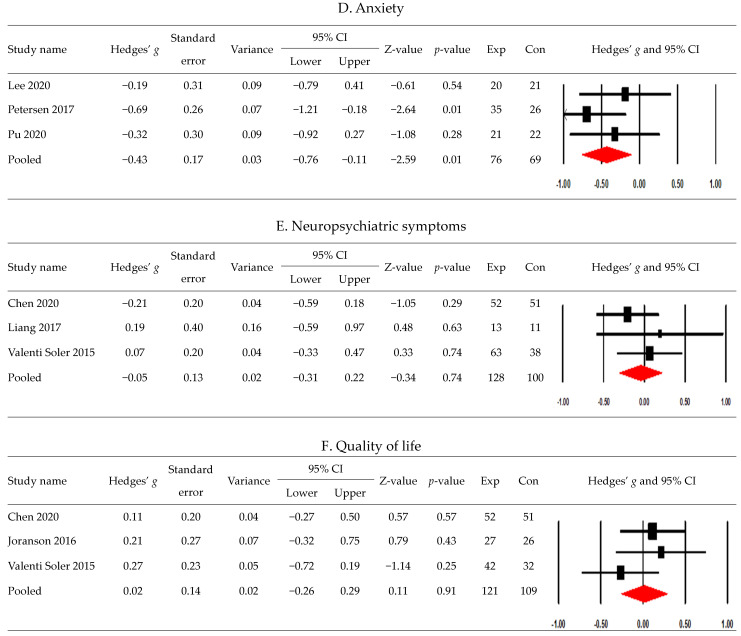
Postintervention effects of robot interventions by the type of outcomes ((**A**) depression; (**B**) cognitive function; (**C**) agitation; (**D**) anxiety; (**E**) neuropsychiatric symptoms; (**F**) quality of life) [15,20,36,37,38,39,41,42,43,44].

Figure 2 displays the effects of robot interventions on depression, cognitive function, agitation, anxiety, neuropsychiatric symptoms, and quality of life.

Among the eight studies assessing depression, seven reported sufficient numerical data and were pooled using a meta-analysis [15,20,36,39,42,43,44], and the results indicated no significant decrease in the intervention group compared with the control group (*g* −0.27; 95% CI, −0.54 to 0.00) and the heterogeneity scores were Tau^2^ = 0.06, Q = 11.19, *p* = 0.08, and *I*^2^ = 46.37%.

Among the six studies assessing cognitive function, five reported sufficient numerical data and were synthesized quantitatively [20,39,41,42,43]. One study used two intervention groups of a pet-type robot and a humanoid robot with a control group and reported the cognitive function values of three groups [41]. Therefore, the summary statistics of the two robot groups were combined into those of a single experimental group according to the Cochrane Handbook for Systematic Reviews of Interventions [23]. The results showed no significant difference between the groups (*g*, 0.04; 95% CI, −0.16 to 0.24) and had unimportant statistical heterogeneity (Tau^2^ = 0.00, Q = 0.94, *p* = 0.92, and *I*^2^ = 0%).

A meta-analysis was performed on the effects of robot interventions on agitation by pooling four studies [20,36,38,44]. The results showed a significant decrease in the intervention group compared with the control group (*g*, −0.31; 95% CI, −0.62 to −0.00), and the heterogeneity scores were Tau^2^ = 0.03, Q = 4.22, *p* = 0.24, and *I*^2^ = 28.92%.

Three studies assessing anxiety were synthesized [15,43,44]; this resulted in a significant decrease in the intervention group compared with the control group (*g*, −0.43; 95% CI, −0.76 to −0.11), with unimportant statistical heterogeneity (Tau^2^ = 0.00, Q = 1.75, *p* = 0.42, and *I*^2^ = 0%).

A meta-analysis pooling three studies showed no significant difference in neuropsychiatric symptoms between the groups (*g*, −0.05; 95% CI, −0.31 to 0.22) and unimportant statistical heterogeneity (Tau^2^ = 0.00, Q = 1.32, *p* = 0.52, and *I*^2^ = 0%) [20,41,42]. One study reported the means and SDs of neuropsychiatric inventory outcomes for two experimental groups (a humanoid robot group and a pet robot group) and one control group [41]; the means, SDs, and sample sizes across the two experimental groups were combined into those of a single experimental group according to the guideline of the Cochrane Handbook for Systematic Reviews of Interventions [21].

A meta-analysis of quality of life synthesizing three studies showed no significant difference between the groups (*g*, 0.02; 95% CI, −0.26 to 0.29) and possible unimportant heterogeneity (Tau^2^ = 0.01, Q = 2.23, *p* = 0.33, and *I*^2^ = 10.45%) [37,41,42].

### 3.5. Postintervention Effects Based on Robot Type

**Figure 3:**
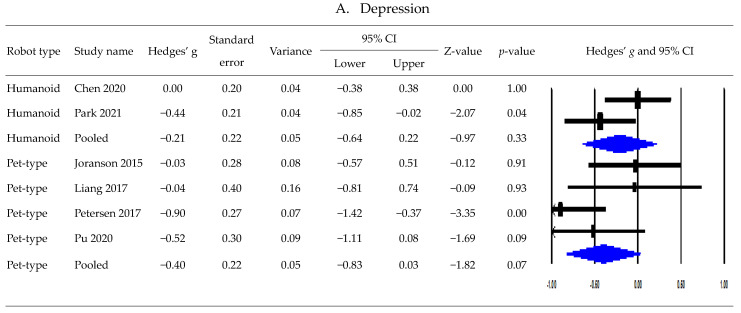

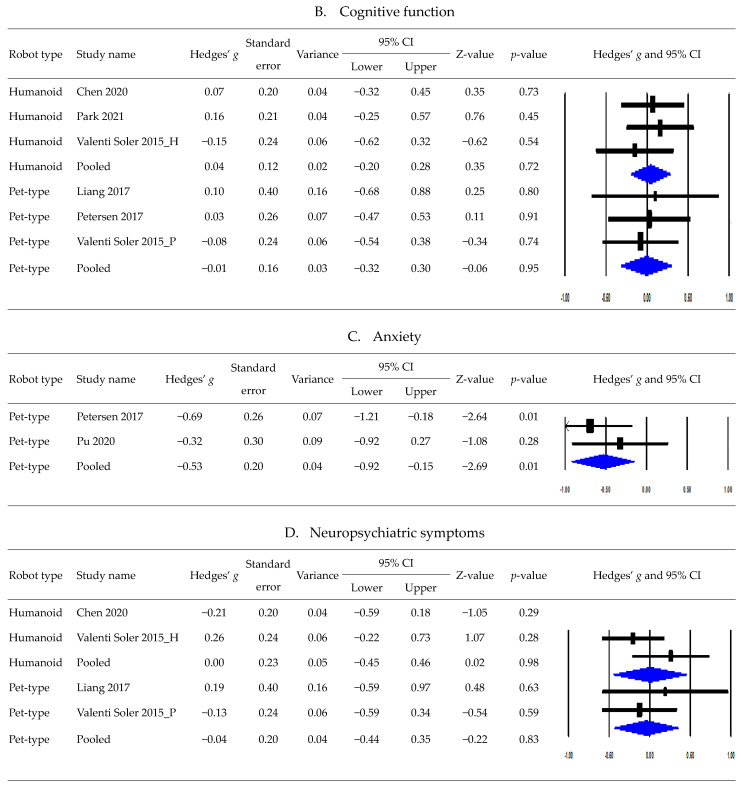

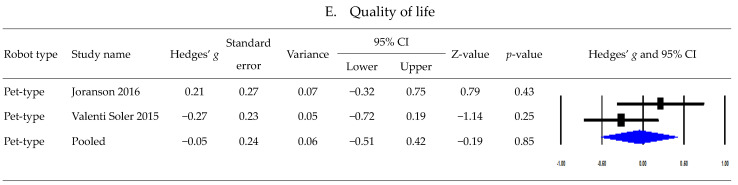
Subgroup analysis results by the type of robots ((**A**) depression; (**B**) cognitive function; (**C**) anxiety; (**D**) neuropsychiatric symptoms; (**E**) quality of life) [20,36,37,39,41,42,43,44].

Figure 3 presents the subgroup meta-analysis results of postintervention effects by type of robot. Regarding the effects of robot interventions on depression according to robot type, a subgroup meta-analysis that pooled four studies using a pet-type robot revealed no significant effect (*g*, −0.40; 95% CI, −0.83 to 0.03; Tau^2^ = 0.10; Q = 6.18; *p* = 0.10; *I*^2^, 51.47%) [20,36,43,44]. An analysis pooling two studies using humanoid robots showed no significant effect (*g*, −0.21; 95% CI, −0.64 to 0.22; Tau^2^ = 0.05; Q = 2.30; *p* = 0.13; *I*^2^, 56.50%) [39,41].

A subgroup meta-analysis of the effects of pet-type robots on cognitive function pooled three studies and showed no significant effect (*g*, −0.01; 95% CI, −0.32 to 0.30; Tau^2^ = 0.00; Q = 0.19; *p* = 0.91; *I*^2^, 0%) [20,41,43]. Additionally, a subgroup analysis pooling three studies revealed no significant difference in cognitive function between the humanoid robot and control groups (*g*, 0.04; 95% CI, −0.20 to 0.28; Tau^2^ = 0.00; Q = 0.96; *p* = 0.62; *I*^2^, 0%) [39,41,42].

Among the three studies assessing anxiety, two used a pet-type robot [43,44], and a subgroup analysis synthesizing the two studies showed that pet-type robot interventions significantly reduced anxiety (*g*, −0.53; 95% CI, −0.92 to −0.15; Tau^2^ = 0.00; Q = 0.85; *p* = 0.36; *I*^2^, 0%).

Among the three studies assessing neuropsychiatric symptoms, one used a pet-type robot [20], another used a humanoid robot [42], and the third used both types of robots [41]. A subgroup meta-analysis of the effects of a pet-type robot pooled two studies and showed no significant difference between the intervention and control groups (*g*, −0.04; 95% CI, −0.44 to 0.35; Tau^2^ = 0.00; Q = 0.47; *p* = 0.49; *I*^2^, 0%) [20,41]. Moreover, an analysis of the effects of humanoid robots on neuropsychiatric symptoms pooling two studies revealed no significant difference between the humanoid robot and control groups (*g*, 0.00; 95% CI, −0.45 to 0.46; Tau^2^ = 0.06; Q = 2.23; *p* = 0.14; *I*^2^, 55.09%) [41,42].

Among the three studies assessing quality of life, one used a pet-type robot [37], another used a humanoid robot [42], and the third study [41] presented only the results on the quality of life using a pet-type robot. The results of a meta-analysis pooling two studies revealed no significant difference between a pet-type robot and control groups (*g*, −0.05; 95% CI, −0.51 to 0.42; Tau^2^ = 0.05; Q = 1.80; *p* = 0.18; *I*^2^, 44.34%) [37,41].

### 3.6. Postintervention Effects According to Intervention Formats

**Figure 4:**
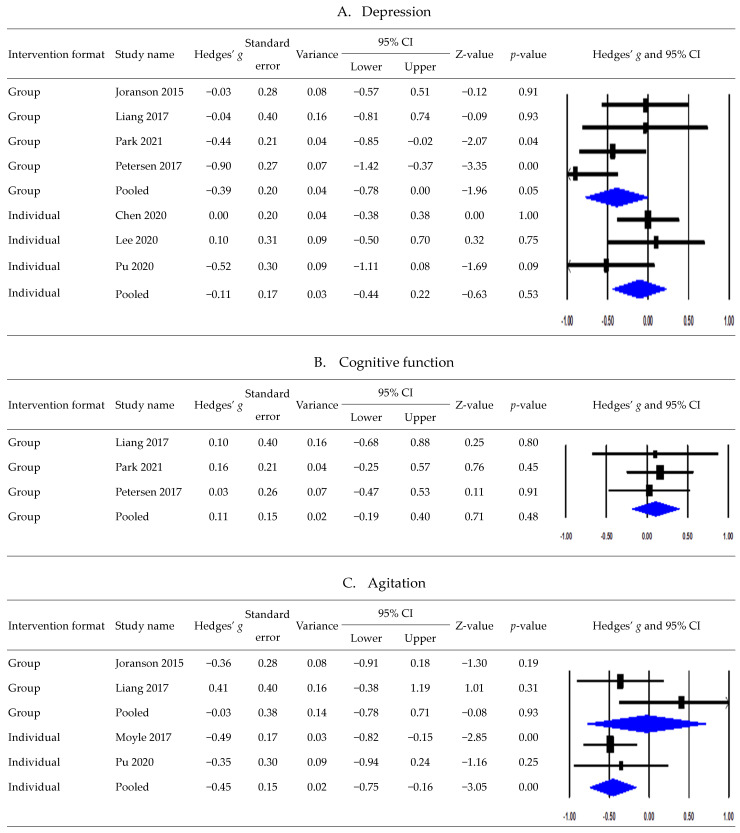

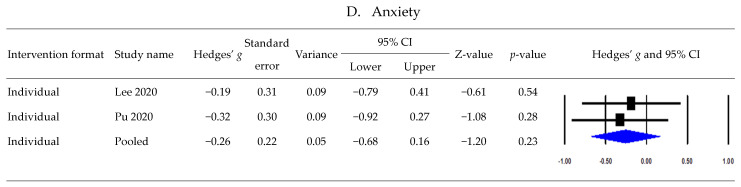
Postintervention effects by intervention formats ((**A**). depression; (**B**). cognitive function; (**C**). agitation; (**D**). anxiety) [15,20,36,38,39,42,43,44].

Figure 4 presents the subgroup meta-analysis results of postintervention effects by intervention format. Regarding the effects of robot interventions on depression according to intervention formats, a subgroup meta-analysis pooling three studies using the individual approach showed no significant difference between the intervention and control groups (*g*, −0.11; 95% CI, −0.44 to 0.22; Tau^2^, 0.02; Q, 2.55; *p* = 0.28; *I*^2^, 21.48%) [15,42,44]. However, an analysis pooling four studies using group-based interventions showed that robot interventions significantly reduced depression (*g*, −0.39; 95% CI, −0.78 to 0.00; Tau^2^, 0.08; Q, 6.08; *p* = 0.11; *I*^2^, 50.69%) [20,36,39,43].

Among the five studies included in the quantitative synthesis of cognitive function, three studies performed group interventions using a robot [20,39,43], and one study applied individual or group intervention formats based on the participants’ dementia severity [41]. Pooling these three studies revealed no significant difference between group-based robot interventions and control conditions (*g*, 0.11; 95% CI, –0.19 to 0.40; Tau^2^, 0.00; Q, 0.16; *p* = 0.92; *I*^2^, 0%) [20,39,43].

Regarding the effects of robot interventions on agitation according to intervention formats, a subgroup analysis pooling two studies that used individual interventions showed a significant reduction (*g*, −0.45; 95% CI, −0.75 to −0.16; Tau^2^, 0.00; Q, 0.16; *p* = 0.69; *I*^2^, 0%) [38,44]. By contrast, an analysis pooling two studies using group-based interventions showed no significant difference between the intervention and control groups (*g*, −0.03; 95% CI, −0.78 to 0.71; Tau^2^, 0.18; Q, 2.48; *p* = 0.12; *I*^2^, 59.69%) [20,36].

In a subgroup meta-analysis synthesizing two studies that employed individual interventions among the three studies assessing anxiety, no significant difference was observed between the groups (*g*, −0.26; 95% CI, −0.68 to 0.16; Tau^2^, 0.00; Q, 0.10; *p* = 0.75; *I*^2^, 0%) [15,32].

## 4. Discussion

This study was designed to examine the effectiveness of robot interventions in improving cognitive and psychological outcomes in older adults with cognitive impairment. We identified 10 studies from 11 papers, and most targeted older adults with dementia, except for two studies that focused on individuals with MCI. The socially assistive robots used in the included studies were largely divided into pet-type and humanoid robots. All studies employing pet-type robots used PARO, which looks similar to a baby harp seal, whereas the studies using humanoid robots used three kinds of robots. Some interventions employed an individual approach or group formats. The most frequently measured outcome in the included studies was depression (measured in eight studies), followed by cognitive function (measured in six studies) and agitation (measured in four studies). Moreover, anxiety, neuropsychiatric symptoms, and quality of life were measured in three studies each.

Regarding the effects of robot interventions on anxiety, the meta-analysis pooling three studies showed that robot interventions had a small, significant effect on decreasing anxiety (*g*, −0.43), and the subgroup meta-analysis pooling two studies showed that pet-type robot interventions had a significant, medium effect on decreasing anxiety (*g*, −0.53). Among the three studies, one reported that robot cognitive interventions using a tabletop-type robot reduced anxiety but stated a limitation of not being sure whether the effect was due to the use of the robots or the cognitive intervention itself [15]. Among the remaining two studies using PARO, one reported a significant decrease in anxiety in the experimental group consisting of individuals who interacted with PARO for three months compared with that of the control group [43]. By contrast, one study reported no significant difference in anxiety between the intervention group consisting of individuals who interacted with PARO for six weeks and the control group. This indicates that the six-week period was inadequate to change an individual’s mood [44]. Overall, this review provides support for a significant reduction in anxiety among older adults with cognitive impairment through robot interventions. However, further studies are required to examine the underlying roles of robot interventions in decreasing anxiety and to determine the optimal duration of interaction with pet-type robots for the anxiety-reducing effect to occur.

All four studies assessing agitation used PARO, and PARO interventions had a small, significant effect in reducing agitation among older adults with cognitive impairment (*g*, −0.31). The subgroup analysis revealed that individual interventions using PARO had an effect with a small effect size on agitation (*g*, −0.45), whereas the effect of group interventions on agitation using PARO was insignificant. Leng et al. [45] suggested that personalized intervention considering an individual’s abilities and needs is important to improve agitation in dementia patients through a review of non-pharmacological interventions. Lu et al. [46] reported that a high degree of personalization in interventions includes a comprehensive assessment and consideration of individual capacity, preferences, interests, and environments in the design and delivery of interventions. Therefore, to reduce the agitation of older adults with cognitive impairment, individual intervention using a robotic pet that includes a high degree of personalization can be considered.

The robot interventions using the individual approach had no significant effect on depression, whereas group interventions had a small effect in reducing depression (*g*, −0.39). These results suggest that robot interventions, especially group-based format, can help decrease depression. Studies that used the individual intervention format discussed reasons for the insignificant effect of robot interventions on decreasing depression. For example, one study using home-based robot cognitive interventions reported that the cognitive intervention was not significantly effective because the participants were not clinically significantly depressed at baseline [15]. Another study that employed PARO using the individual intervention format reported that interactions with the robotic pet for 6 weeks were not significantly effective because the intervention period was inadequate to elicit mood changes [44].

Regarding the effects of robot interventions on cognitive function, neuropsychiatric symptoms, and quality of life, the meta-analyses showed no significant differences between the experimental and control groups. Regarding the effects of robot interventions on cognitive function, one study reported that robot interventions did not improve cognitive function because most participants had severe dementia [42]. In contrast, a study targeting MCI participants or participants with subjective memory complaints reported that robot interventions significantly affected cognitive function [39]. Consistently, a study found that the cognitive level of participants who expressed a positive feeling toward PARO was significantly higher than that of participants who expressed negative feelings toward the robot, suggesting that robot interventions are effective for participants with MCI who respond better to robots compared with those with severe cognitive impairment [20]. However, only two studies targeted older adults with MCI; hence, further studies are needed to identify the effectiveness of robot interventions in improving cognitive function among older adults with MCI.

All three studies assessing neuropsychiatric symptoms found no significant difference between the experimental and control groups. However, one study suggested that robot interventions may reduce neuropsychiatric symptoms based on findings that the level of neuropsychiatric symptoms of the experimental group decreased during the robot intervention period; nonetheless, the level of neuropsychiatric symptoms returned to the baseline level during the withdrawal phase after the removal of the robot [42]. Furthermore, one study reported that robot interventions significantly affected some neuropsychiatric symptoms, such as irritability, delusions, and apathy, although the total score was insignificant [41]. Given this evidence, robot interventions could reduce neuropsychiatric symptoms among older adults with cognitive impairment. However, identifying the significant effects of robot interventions on overall neuropsychiatric symptoms is difficult because the tool for assessing neuropsychiatric symptoms consists of 12 sub-symptoms.

Regarding the effects of robot interventions on quality of life, two studies revealed no significant difference between the experimental and control groups [37,42], and one study reported that the quality of life of the robot intervention group was worse than that of the control group receiving conventional therapy [41]. Therefore, the effectiveness of robot interventions in improving quality of life cannot be compared because of insufficient data. Quality of life is important for people with cognitive impairment for the rest of their lives [47]. However, only three studies in this review assessed quality of life as an outcome, which is similar to that presented in a previous scoping review [47]. In future studies that will evaluate the effectiveness of robot interventions, it is necessary to include quality of life as an outcome and reanalyze the integrated results of intervention effects on quality of life.

### Limitations

This review had some limitations. First, this review restricted the search to articles reported in English. Second, despite the extensive search, only 10 studies met the inclusion criteria. Although two or more studies are sufficient for a meta-analysis [26], future meta-analyses combining further RCTs are needed to provide more robust evidence. Third, only two studies targeting those with MCI were included in this review. Thus, data from older adults with MCI are needed to identify the effectiveness of robot interventions in this patient population.

## 5. Conclusions

This review showed that robot interventions significantly decrease anxiety and agitation in older adults with cognitive impairment. However, given that this review showed that robot interventions had no significant effect on improving cognitive function, further research is needed to determine whether robot interventions affect cognition. Additionally, a pet-type robot effectively reduced anxiety and agitation in older adults with cognitive impairment. Humanoid robots were employed in only three studies, indicating a lack of research on the effectiveness of humanoid robots; thus, further research is necessary. Although subgroup analyses according to intervention formats showed that individual interventions reduce agitation and group-based interventions reduce depression, further research is needed to compare the effects of the individual approach with those of the group approach.

## Figures and Tables

**Table 1:** Detailed descriptions of reviewed articles.

Author/Country	Study Design	Participants’ Characteristics (Sample Size, Settings and Cognitive Status, Age Range, Mean or Median Age, and Sex)	Intervention Characteristics (Intervention Method, Duration, Frequency, and Time/Session)	Comparison	Outcomes Related to Cognitive and Psychological Status (Measurement Tools)	Measurement Assessment Time Points
Chen et al. [42]/Hong Kong	RCT with ABAB withdrawal design	103 (E: 52, C: 51); long-term care facility residents with dementia; aged 67–108 years; mean age, 87.2 years; 79.6% were women	Individual, unfacilitated intervention with a humanoid robot (Kabochan); usual care for 8 weeks (baseline phase), then intervention for 8 weeks (intervention phase), then usual care for 8 weeks (intervention-withdrawal phase), and then intervention for 8 weeks (intervention-reintroduction phase)	Usual care	Cognitive function (Montreal Cognitive Assessment 5 min Protocol) Depression (Geriatric Depression Scale) Neuropsychiatric symptoms (Neuropsychiatric Inventory Questionnaire) Quality of life (Quality of Life in Alzheimer’s Disease Scale)	Week 1 (baseline), week 8 (baseline), week 16 (post-first intervention), week 24 (withdrawal), and week 32 (post-second intervention)
Jøranson et al. [36]/Norway Jøranson et al. [37]/Norway	CRT	53 (E: 27, C: 26); residents with mild to severe dementia in nursing home; aged 62–95 years; mean age, 84.0 years; 67% were women	Group activity with the seal robot (PARO) delivered in nursing homes; for 12 weeks, twice a week; each session lasted 30 min	Treatment as usual	Agitation (Brief Agitation Rating Scale) Depression (Cornell Scale for Depression in Dementia) Quality of life (Quality of Life in Late-Stage Dementia Scale)	Baseline, postintervention, and 3 months after the end of intervention (follow-up)
Lee et al. [15]/Korea	RCT	41 (E: 20, C: 21); MCI patients recruited from a hospital; aged 60 years or older; mean age, 74.0 years; 39.1% were women	Home-based cognitive intervention with a personal robot (Bomy); for 4 weeks, 5 days a week; each session lasted 60 min	No intervention	Anxiety (Geriatric Anxiety Inventory) Depression (Geriatric Depression Scale-Short Form) Spatial working memory, paired-associates learning, rapid visual information processing (Cambridge Neuropsychological Test Automated Battery)	Baseline and postintervention
Liang et al. [20]/New Zealand	RCT	24 (E: 13, C: 11); participants with dementia who attended dementia daycare centers; aged 67–98 years; mean age, 83.8 years; 64% were women	Group activity with a seal robot (PARO) at daycare centers and interactions with the robot at home; for 6 weeks, 2–3 times a week; each session lasted 30 min	Standard care	Agitation (Cohen–Mansfield Agitation Inventory-Short Form) Cognitive function (Addenbrooke’s Cognitive Examination) Depression (Cornell Scale for Depression in Dementia) Neuropsychiatric symptoms (Neuropsychiatric Inventory Brief Questionnaire Form)	Baseline, 6 weeks (postintervention), and 12 weeks (follow-up)
Moyle et al. [38]/Australia	CRT	415 (E: 138, C1: 140, C2: 137); long-term care facility residents with dementia; aged 60 years or older; mean age, 85.0 years; 75.7% were women	Individual, unfacilitated sessions with a robotic seal (PARO); for 10 weeks, thrice a week; each session lasted 15 min	C1: nonrobotic plush toy, C2: usual care	Agitation (Cohen–Mansfield Agitation Inventory-Short Form) Mood states (Coded video observations)	Baseline, week 1, week 5, week 10 (postintervention), and week 15 (follow-up)
Park et al. [39]/Korea	RCT	135 (E: 45, C1: 45, C2: 45); community-dwelling older adults with MCI or subjective memory complaints recruited from a dementia center; aged 60 years or older; mean age, 75.9 years; 72.6% were women	Robot-assisted cognitive training group program (humanoid robot, Sil-bot) delivered in a dementia center; for 6 weeks, twice a week; each session lasted 60 min	C1: traditional cognitive training, C2: no intervention	Cognitive function (Mini-Mental State Examination-Dementia Screening) Depression (Geriatric Depression Scale-Short Form) Neuropsychological assessment (Consortium to Establish a Registry for Alzheimer’s Disease) Subjective memory complaints (Subjective Memory Complaint Questionnaire)	Baseline and postintervention
Peterson et al. [43]/USA	RCT	61 (E: 35, C: 26); long-term care facility residents with mild to moderate dementia; aged 65 years or older; mean age, 83.4 years; 77.0% were women	Group sessions with a robotic pet (PARO); for 3 months, thrice a week; each session lasted 20 min	Standard care	Anxiety (Rating Anxiety in Dementia) Cognitive function (Global Deterioration Scale) Depression (Cornell Scale for Depression in Dementia)	Baseline and postintervention
Pu et al. [44]/Australia	RCT	43 (E: 21, C: 22); long-term care facility residents with chronic pain and dementia; aged 65–97 years; mean age, 86.0 years; 69.8% were women	Individual, unfacilitated sessions with a robotic seal (PARO); for 6 weeks, 5 days a week; each session lasted 30 min	Usual care	Agitation (Cohen–Mansfield Agitation Inventory-Short Form) Anxiety (Rating Anxiety in Dementia) Depression (Cornell Scale for Depression in Dementia)	Baseline and postintervention
Thodberg et al. [33]/Denmark	RCT	100 (E1: 35, E2: 35, C: 30); nursing home residents with dementia; age 79–90 years; median age, 85.5 years; 69% were women	E1: Visits from a person accompanied by a dog, E2: visits from a person accompanied by a robot seal (PARO); for 6 weeks; biweekly; each visit lasted 10 min	Soft toy cat	Cognitive function (Mini-Mental State Examination) Dementia symptoms (Gottfries–Bråne–Steen scale) Depression (Geriatric Depression Scale) Symptoms of delirium (Confusion Assessment Method)	The week before and after the visit period
Valentí Soler et al. [41]/Spain	RCT	Phase 1: 101 (E1: 30, E2: 33, C: 38) Phase 2: 110 (E1: 42, E2: 36, C: 32); nursing home residents with dementia; aged 58–100 years (Phase 1), aged 59–101 years (Phase 2); mean age, 84.7 years (Phases 1 and 2); 88% were women in Phase 1; 90% were women in Phase 2	Phase 1: E1: humanoid robot (NAO), E2: pet robot (PARO) Phase 2: E1: pet robot (PARO), E2: real animal (dog); for 3 months; 2 days a week; each session lasted 30–40 min	Standard care	Apathy (Apathy Scale for Institutionalized Patients with Dementia Nursing Home version) Cognitive function (Mini-Mental State Examination, Severe Mini-Mental State Examination) Neuropsychiatric symptoms (Neuropsychiatric Inventory) Quality of life (Quality of Life in Late-Stage Dementia Scale)	Baseline and postintervention

RCT = Randomized Controlled Trial; CRT = Cluster Randomized Trial.

## Data Availability

The original contributions generated for the study are included in the article/Supplementary Material. Further inquiries can be directed to the corresponding author.

## Supplementary Materials

The following supporting information can be downloaded at: https://www.mdpi.com/article/10.3390/healthcare11162341/s1, Figure S1: Estimated risk of bias across all included studies; Table S1: Search strategies for databases.
