# Supplementary material for: Effectiveness of Robot Interventions for Cognitive and Psychological Outcomes among Older Adults with Cognitive Impairment: A Meta-Analysis
Source: Healthcare (Basel). 2023 Aug 19;11(16):2341. doi: 10.3390/healthcare11162341 (PMC10454070; doi:10.3390/healthcare11162341)
Supplement: Supplementary file 1 [file healthcare-11-02341-s001.zip › healthcare-2518815-supplementary.pdf]

Supplementary materials

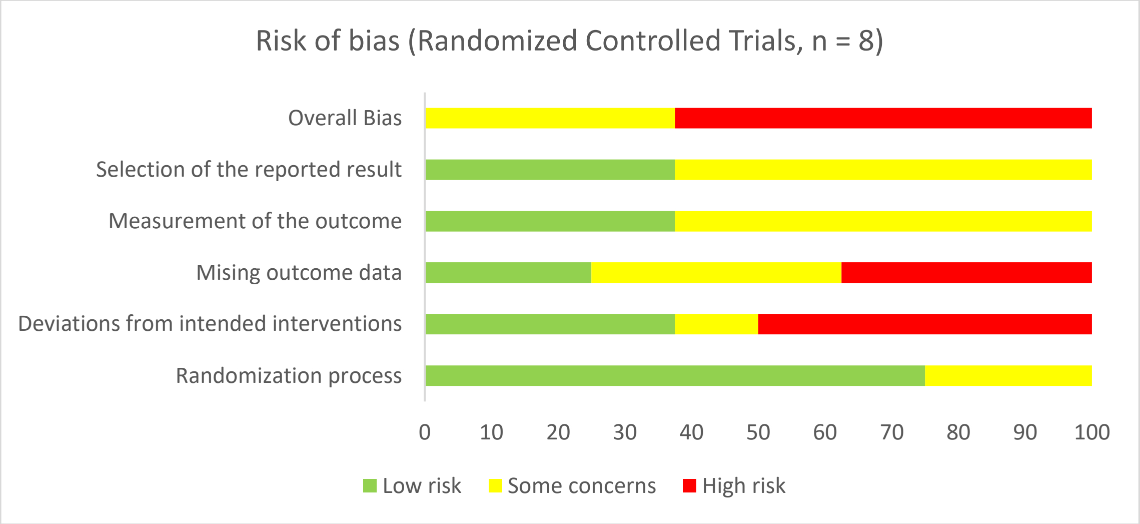

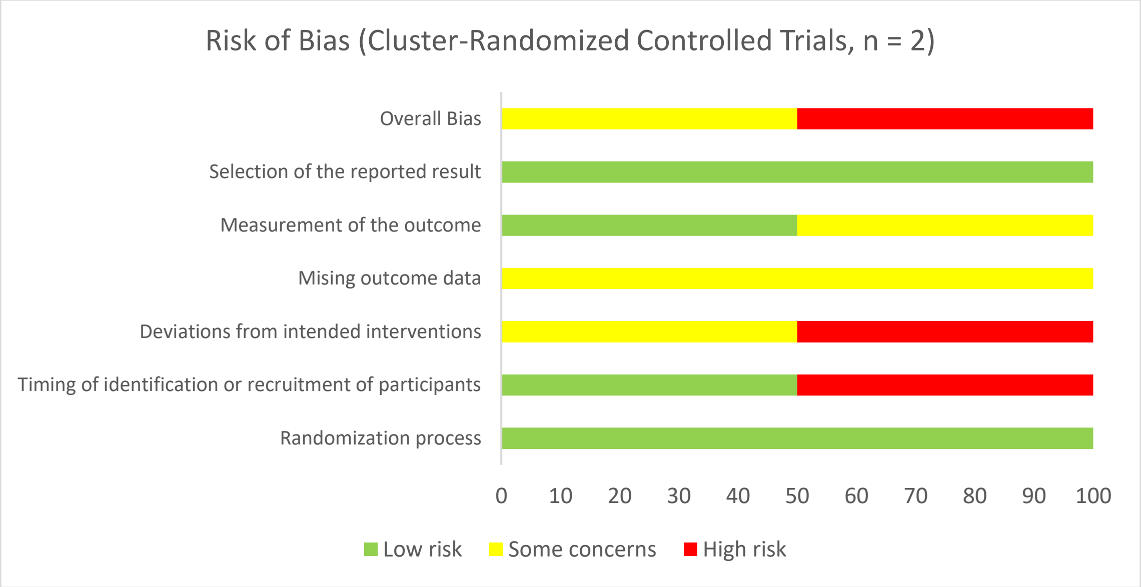

Figure S1. Estimated risk of bias across all included studies

Table S1. Search strategies for databases

No.	Search strategies	Filters	Results
PubMed			
#1	(((((((((cognitive impairment[Title/Abstract]) OR (mild cognitive[Title/Abstract])) OR (MCI[Title/Abstract])) OR (cognitive decline[Title/Abstract])) OR (benign senescent forgetfulness[Title/Abstract])) OR (memory impairment[Title/Abstract])) OR (memory decline[Title/Abstract])) OR (dementia[Title/Abstract])) OR (Alzheimer[Title/Abstract])) OR "Dementia"[Mesh]		336,411
#2	(robot*[Title/Abstract]) OR ("Robotics"[Mesh])		59,019
#3	(randomized controlled trial[pt] OR controlled clinical trial[pt] OR randomized[tiab] OR randomised[tiab] OR placebo[tiab] OR clinical trials as topic[mesh:noexp] OR randomly[tiab] OR trial[ti] NOT (animals[mh] NOT humans [mh]))		1,331,257
#4	#1 AND #2 AND #3		56
#5	#1 AND #2 AND #3	From 2015 - 2021	47
#6	#1 AND #2 AND #3	English	47
Total			47

No.	Search strategies	Filters	Results
Embase			
#1	'cognitive impairment':ab,ti OR 'mild cognitive':ab,ti OR mci:ab,ti OR 'cognitive decline':ab,ti OR 'benign senescent forgetfulness':ab,ti OR 'memory impairment':ab,ti OR 'memory decline':ab,ti OR dementia:ab,ti OR alzheimer:ab,ti		414,775
#2	'dementia'/exp OR 'mild cognitive impairment'/exp		398,577
#3	#1 OR #2		534,640
#4	robot*:ab,ti		79,691
#5	'robotics'/exp		42,441
#6	#4 OR #5		87,374
#7	'randomized controlled trial'/exp		675,523
#8	'controlled clinical trial'/exp		850,097
#9	'clinical trial (topic)'/mj		11,716
#10	randomized:ab,ti OR randomised:ab,ti OR placebo:ab,ti OR randomly:ab,ti OR trial:ti		1,583,554
#11	#7 OR #8 OR #9 OR #10		1,836,936
#12	'animal'/exp		29,445,679
#13	'human'/exp		23,786,146

#14	#12 NOT #13		5,659,533
#15	#11 NOT #14		1,668,664
#16	#3 AND #6 AND #15		75
#17	#3 AND #6 AND #15	From 2015 – 2021, English	57
Total			57

No.	Search strategies	Filters	Results
Cochrane central			
#1	MeSH descriptor: [Dementia] explode all trees		6,231
#2	(cognitive impairment):ti,ab,kw		12,381
#3	(mild cognitive):ti,ab,kw		7,597
#4	(MCI):ti,ab,kw		2,723
#5	(cognitive decline):ti,ab,kw		4,964
#6	(benign senescent forgetfulness):ti,ab,kw		2
#7	(memory impairment):ti,ab,kw		4,835
#8	(memory decline):ti,ab,kw		2,071
#9	(dementia):ti,ab,kw		13,665
#10	(Alzheimer):ti,ab,kw		11,641
#11	#1 OR #2 OR #3 OR #4 OR #5 OR #6 OR #7 OR #8 OR #9 OR #10		34,722
#12	(robot*):ti,ab,kw		5,402
#13	MeSH descriptor: [Robotics] explode all trees		956
#14	#12 OR #13		5,402
#15	MeSH descriptor: [Randomized Controlled Trial] explode all trees		119
#16	MeSH descriptor: [Controlled Clinical Trial] explode all trees		128
#17	(randomized):ti,ab,kw		960,191
#18	(randomised):ti,ab,kw		960,191
#19	(placebo):ti,ab,kw		321,949
#20	(randomly):ti,ab,kw		264,739
#21	(trial):ti		343,973
#22	MeSH descriptor: [Clinical Trials as Topic] this term only		33,265
#23	#15 OR #16 OR #17 OR #18 OR #19 OR #20 OR #21 OR #22		1,204,024
#24	MeSH descriptor: [Animals] explode all trees		614,391
#25	MeSH descriptor: [Humans] explode all trees		614,332
#26	#24 NOT #25		59
#27	#23 NOT #26		1,203,991
#28	#11 AND #14 AND #27		84
#29	#11 AND #14 AND #27	Trials	81
#30	#11 AND #14 AND #27	2015 to 2021	71

Total	71
